# Application of Green Algal *Planktochlorella nurekis* Biomasses to Modulate Growth of Selected Microbial Species

**DOI:** 10.3390/molecules26134038

**Published:** 2021-07-01

**Authors:** Leszek Potocki, Bernadetta Oklejewicz, Ewelina Kuna, Ewa Szpyrka, Magdalena Duda, Janusz Zuczek

**Affiliations:** 1 Department of Biotechnology, College of Natural Sciences, University of Rzeszow, Pigonia 1, 35-310 Rzeszow, Poland; b.oklejewicz@gmail.com (B.O.); gawel.ewelina@gmail.com (E.K.); ewaszpyrka@interia.pl (E.S.); 2 Bioorganic Technologies sp. z o.o., Sedziszow Malopolski, Sielec 1A, 39-120 Sielec, Poland; mduda@bioorganictechnologies.pl (M.D.); janusz@bioorganictechnologies.pl (J.Z.)

**Keywords:** microalgae, *Planktochlorella nurekis*, antibacterial activity, natural preservatives

## Abstract

As microalgae are producers of proteins, lipids, polysaccharides, pigments, vitamins and unique secondary metabolites, microalgal biotechnology has gained attention in recent decades. Microalgae can be used for biomass production and to obtain biotechnologically important products. Here, we present the application of a method of producing a natural, biologically active composite obtained from unicellular microalgae of the genus *Planktochlorella* sp. as a modulator of the growth of microorganisms that can be used in the cosmetics and pharmaceutical industries by exploiting the phenomenon of photo-reprogramming of metabolism. The combination of red and blue light allows the collection of biomass with unique biochemical profiles, especially fatty acid composition (Patent Application P.429620). The ethanolic and water extracts of algae biomass inhibited the growth of a number of pathogenic bacteria, namely *Enterococcus faecalis*, *Staphylococcus aureus* PCM 458, *Streptococcus pyogenes* PCM 2318, *Pseudomonas aeruginosa*, *Escherichia coli* PCM 2209 and *Candida albicans* ATCC 14053. The algal biocomposite obtained according to our procedure can be used also as a prebiotic supplement. The presented technology may allow the limitation of the use of antibiotics and environmentally harmful chemicals commonly used in preparations against *Enterococcus faecalis*, *Staphylococcus aureus*, *Streptococcus pyogenes*, *Pseudomonas aeruginosa*, *Escherichia coli* or *Candida* spp.

## 1. Introduction

Antimicrobial resistance is one of the major public health problems of the 21st century, threatening the effective prevention and treatment of an increasing number of infections caused by pathogenic microorganisms [1]. The overuse of antibiotics, but also their inappropriate use (inappropriate choice, wrong dosage, their use in the treatment of viral infections or their use for zootechnical reasons), contributes to the emergence and spread of multidrug-resistant (MDR) microorganisms [2]. In addition, efforts by numerous researchers to discover new antimicrobial compounds have encountered countless challenges and, in many cases, have failed. In recent decades, there has been a great trend in the research of microalgae, as well as other marine microorganisms, as an alternative source of antibiotics and preservatives. This is important because microalgae have evolved in extremely competitive environments and have had to develop different tolerance and defense strategies against pathogenic microorganisms, predation, herbivory and competition for space in order to survive. The variety of defense mechanisms results in microalgae producing a large pool of diverse biologically active compounds synthesized from different metabolic pathways [3,4]. Moreover, it is indicated that the diversity of microalgae cellular building blocks, mainly cell wall components (polysaccharides, lipids, proteins), whose amount depends on the ecotypes of a given species developing in specific environmental conditions, may affect the biological activity of biomasses obtained from algal cultures. It has been documented that the increased presence of amino acids, terpenoids, phlorotannins, acrylic acid, phenolic compounds, steroids, halogenated ketones and alkanes, cyclic polysulphides, fatty acids and other active compounds in microalgae cells increases their antimicrobial activity. In the case of marine algae, it was shown that increased antimicrobial activity correlated with the presence of acrylic acid. However, it should be emphasized that a serious defect in biomasses isolated from marine microalgae, as opposed to freshwater algae, is their ability to accumulate heavy metal elements [5].

The antimicrobial activity of biomasses obtained from microalgae cultures is widely documented in the scientific literature [6,7]. However, despite much research in this area, there is a need to source new natural biologically active biomasses from algae because of the increasing problem of drug resistance observed among microorganisms. There are literature data indicating the specific antimicrobial properties of individual algal species. Here we should mention the best-studied microalgae species *Chlorella vulgaris*, which shows the ability to produce chlorelin, a mixture of fatty acids (FAs) inhibiting the growth of G (+) and G (−) bacteria [4,8]. Moreover, the biomass of other algal species, such as *Chlamydomonas reinwardti*, *Chroococcus dispersus*, *Spirulina platensis*, *Chlorella pyrenoidos*, *Dunaliella* sp., *Cenedesmus obliquus* and *Nostoc muscorum*, exhibit not only antibacterial, antiviral and antifungal, but also antioxidant, anticancer, anti-inflammatory and anti-allergic activities [9,10,11,12,13,14]. From a microbiological point of view, it is particularly desirable to obtain algal biomasses which, due to differences in their biochemical profile, are able to inhibit the survival of pathogenic microorganisms and, at the same time, promote the growth of probiotic bacteria which are beneficial to the proper functioning of human and animal microbiota. In previous studies, metabolic reprogramming using a combination of red and blue light of the green alga *Planktochlorella nurekis* (PN) allowed biomasses with a unique biochemical profile, especially fatty acid composition, to be obtained [15].

The aim of this study was to evaluate the biological activity of biomasses obtained by photoprogramming the metabolism of single-celled microalgae of the genus *Planktochlorella nurekis* as a modulator of microbial growth. Additionally, the prebiotic effect of the investigated biomasses on the growth of probiotic species *Lactobacillus rhamnosus* ATCC 53103 was evaluated for the first time, as well as synergistic and antagonistic effects of the obtained extracts on the growth of microorganisms in mixed cultures.

## 2. Results and Discussion

### 2.1. Antimicrobially Active Ethanolic Extracts of the Microalga Planktochlorella nurekis 

**Figure 1:**
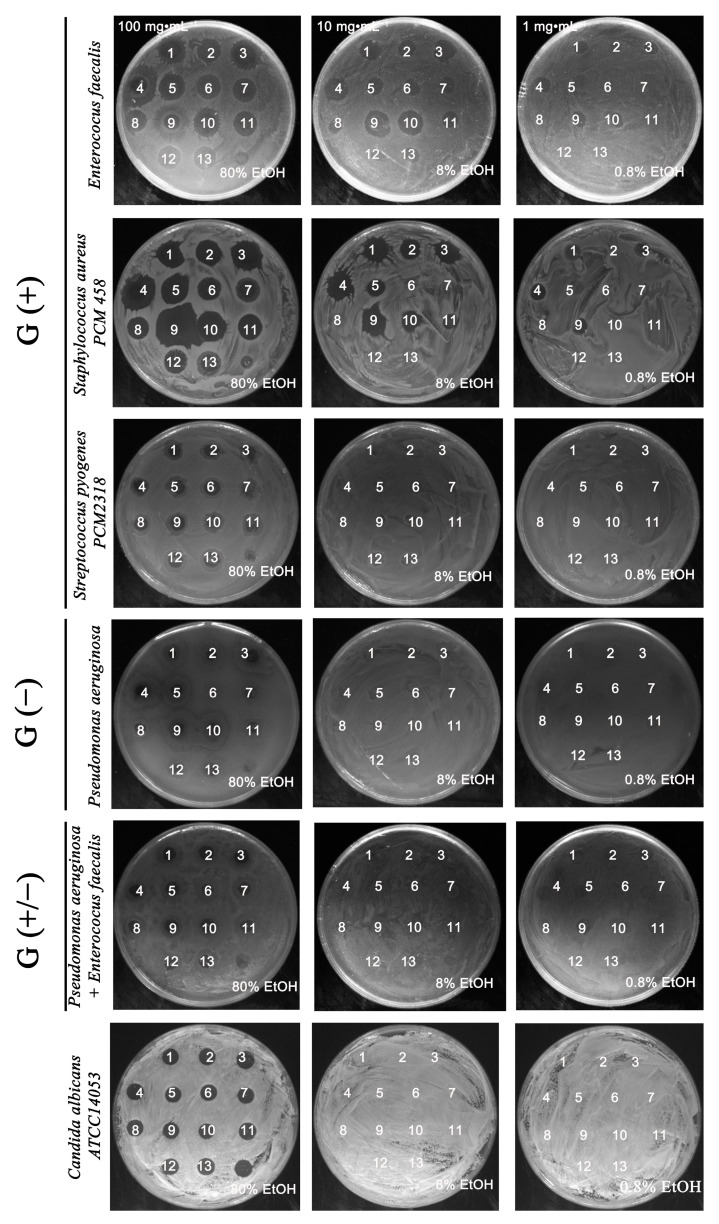
Ethanolic extract-mediated changes (1–13) on microbial growth: G (+) Enterococcus faecalis, Staphylococcus aureus PCM458, Streptococcus pyogenes PCM2318; G (−) Pseudomonas aeruginosa; mixed (G (+/−)) Pseudomonas aeruginosa + Enterococcus faecalis, Candida albicans ATCC14053. The tested microorganisms were treated with microalgae ethanolic extracts (1–13) at 100 mg·mL^−1^, 10 mg·mL^−1^ and 1 mg·mL^−1^ surface spotted onto the indicator lawn—NA agar (bacteria) and YPD agar (Candida) media. Representative micrographs of bacterial and yeast culture dishes.

In the present study we focused on the in vitro evaluation of the antimicrobial properties of biomasses obtained from twelve clones of the microalga *Planktochlorella nurekis* with improved biochemical profiles based on changes in ploidy state and DNA content under the influence of colchicine and cytochalasin B, described in previous work [15]. The antimicrobial activity of the prepared aqueous and ethanolic extracts (1–13 WE, EE) was tested against selected bacterial species, as well as the yeast *Candida albicans* ATCC 14053. Due to rapid drug resistance, these microorganisms are very often the cause of hospital infections. *Staphylococcus* is one of the bacterial species that is most rapidly resistant to the therapeutic agents used and is the etiological agent of many human and animal diseases. These bacteria are responsible for skin infections that are difficult to treat and may eventually lead to the deaths of patients suffering from infected wounds [16,17]. In addition, numerous literature data confirm the increase in infections with vancomycin-resistant *Enterococcus faecalis*, *Pseudomonas aeruginosa* or multidrug-resistant *Candida albicans* strains [18,19,20]. The occurrence of multi-drug resistance in *Escherichia coli* is also a matter of concern, due to the high capacity of this species to accumulate resistance genes, mainly through horizontal gene transfer, which can then be passed on to other bacterial species [21]. There is therefore an urgent need to search for new substances that can be used as antimicrobial drugs. Due to their metabolic plasticity, microalgae represent a promising alternative to fight pathogenic microorganisms [22]. Microbiological analysis of *Planktochlorella nurekis* biomasses, characterized by unique biochemical composition, showed increased antimicrobial activity of ethanol extracts using a spot-on-lawn method. The initial screening identified 11 algal *Planktochlorella nurekis* strains whose ethanol extracts had an antimicrobial activity on the growth of G (+) bacteria, i.e., *Enterococcus faeaclis* and *Staphylococcus aureus*, in a concentration of 10 mg·mL^−1^. In particular, the strongest bactericidal effect was observed for the extracts obtained from biomass 9 > 10 > 4 > 3 > 1. In the case of *Staphylococcus aureus*, the bactericidal effect on growth was observed for extracts 1, 2, 3, 4, 5, 9, 10 at a concentration of 10 mg·mL^−1^, with the strongest bactericidal effect shown by extracts 9 > 4 > 1 (concentration 10 mg·mL^−1^) against extract 13, obtained from the starting wild-type strain *Planktochlorella nurekis* (Figure 1). In the case of *Pseudomonas aeruginosa* G (−), mixed bacteria cultures G (+/−), and yeast *Candida albicans* ATCC 14053, no inhibitory effect of 10 mg·mL^−1^ and 1 mg·mL^−1^ ethanolic extracts on the growth of microorganisms was demonstrated (Figure 1). Furthermore, water extracts obtained from tested biomasses with a concentration of 100 mg·mL^−1^, 10 mg·mL^−1^ and 1 mg·mL^−1^ did not show an inhibitory effect on the growth of tested microorganisms in the spot-on-lawn method.

The extracts used in this study had stronger antibacterial than antifungal activities. The analyses indicate the different susceptibilities of bacteria, i.e., Gram-negative bacteria were insensitive in contrast to Gram-positive bacteria to the used ethanolic extracts. The difference between Gram-positive and Gram-negative bacteria is related to the constructions of the cell wall. Gram-negative bacteria have a more complex structure of the cell wall, which, apart from the peptidoglycan, also contains an outer membrane with lipopolysaccharides LPS, and lipoproteins [23,24]. This extra membrane acts as a „molecular filter”, effectively protecting bacterial cells from the ingress of antibiotics and other chemicals [25]. This outer membrane function of Gram-negative bacteria can explain their lack of sensitivity to the used algal extracts, in contrast to Gram-positive bacteria. It seems that the poor antifungal activity may also be due to the structure and poor permeability of the yeast cell wall containing polysaccharides such as chitin and glucan [26,27]. Additionally, the variation in the recorded activity of the tested algal extracts was probably linked with the type of extraction solvent. This may indicate that some antimicrobial active substances contained in *Planktochlorella nurekis* algal biomasses dissolve to different degrees in the used solvents. The literature review indicates that there are no general recommendations for methods of extraction of biologically active substances from micro- and macroalgae. The yield of extractable substances and antimicrobial agents from different algal species depends on the solvent used. Its choice may in turn depend on the target metabolites released during the extraction process from algal biomasses, as well as the target microorganism species [28]. Our results are consistent with other findings indicating ethanol extracts as the most effective agent against many bacterial species. Karthikeyan et al. (2015) indicates that ethanolic extracts of seaweeds *Enteromorpha* sp., *Cystoseria indica*, *Sargassum swartzii*, *Gracilaria corticata* and *Caulerpa taxifolia* showed antimicrobial activity against Gram-positive and Gram-negative bacteria, namely *Escherichia coli*, *Proteus* sp., *Pseudomonas aeruginosa*, *Klebsiella pneumoniae* and *Staphylococcus aureus* [29]. The most effective ethanolic extracts with in vitro antimicrobial activity against *Streptococcus pyogenes*, *Staphylococcus aureus*, *Listeria monocytogenes*, *Escherichia coli O157: H7*, *Salmonella* spp. were also obtained from biomasses of the algae *Scenedesmus obliqus*, *Chlorella vulgaris*, *Nostoc* sp., *Pithophora oedogonium*, *Dunaliella salina* or *Myagropsis myagroides* [30,31]. Furthermore, the results obtained in our study confirm the observations of other researchers, indicating that antimicrobial activity may vary among ecotypes of the same algal species adapting to different environmental conditions, their concentration and the tested microorganisms [32,33].

### 2.2. Different Effects (Stimulatory and Inhibitory) of Water and Ethanolic Extracts (WE, EE) on Metabolic Activity of the Studied Microorganisms

**Figure 2:**
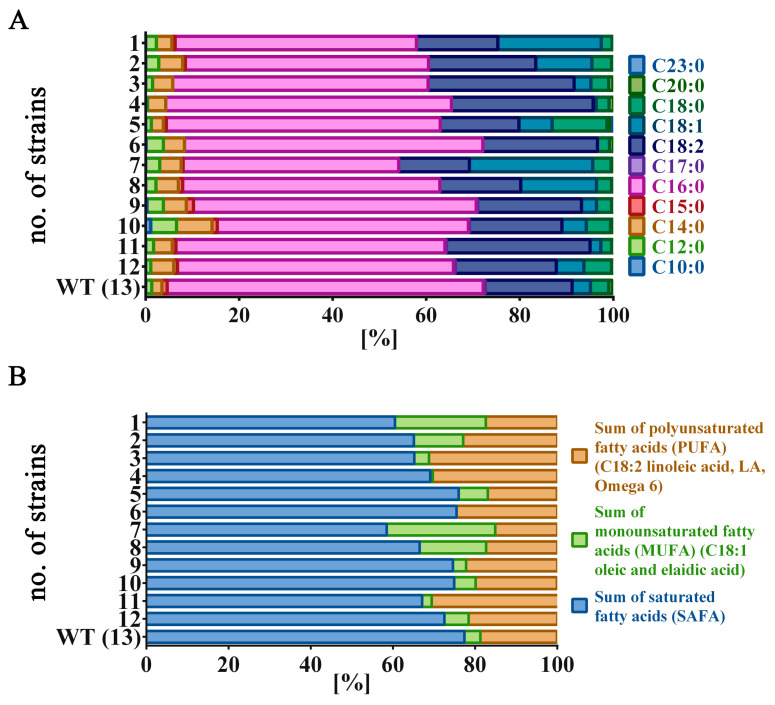
Fatty acid profiles of *Planktochlorella nurekis* strains (1–13). (**A**) distribution of fatty acids in biomass of individual *Planktochlorella nurekis* strains; (**B**) Major fatty acid groups of *Planktochlorella nurekis* strains (1–13): saturated fatty acids (SAFA), monounsaturated fatty acids (MUFA), polyunsaturated fatty acids (PUFA). Percentage contents of FAs are shown.

After preliminary analyses of antimicrobial activity, the effect of the analyzed water and ethanolic (WE, EE) extracts on the metabolic activity of selected bacterial species, as well as *Candida albicans*, was further examined using a resazurin test. The obtained results are described and discussed with consideration as an analysis of the correlation between the content of some lipid fractions, namely saturated fatty acids (SAFA), monounsaturated fatty acids (MUFA), polyunsaturated fatty acids (PUFA) and selected fatty acids (determined in biomasses of *P. nurekis* in our previously study) with the metabolic activity of the investigated microorganisms Figure 2A,B) [15]. The fatty acids, which constitute major parts of the extracted *P. nurekis* biomasses, are particularly considered because their antimicrobial effects have been long recognized [11,34]. In the literature, fatty acids, which constitute the main fraction of the ethanolic or hexane extract of *Dunaliella salina*, have been described as responsible for antimicrobial properties against *E. coli*, *S. aureus* and *C. albicans* [35]. Additionally, the antimicrobial activity of algal species: *Nostoc spongiforme*, *Oscillatoria tenius* and *Chlorococcus* sp., were associated with their fatty acid content [36,37].

**Figure 3:**
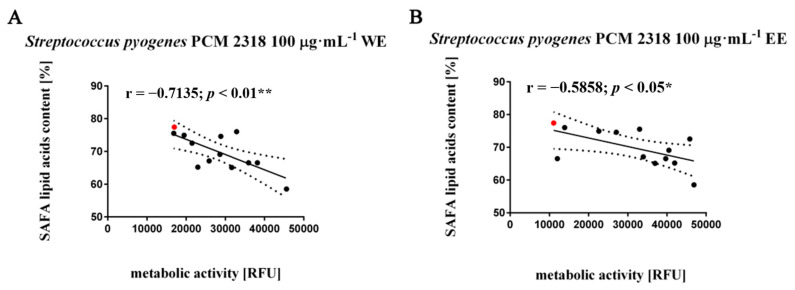
Effect of saturated fatty acids (SAFA) (**A**,**B**); on metabolic activity of *Streptococcus pyogenes* PCM 2318. Twelve water or ethanol extracts with defined concentrations of 1 mg·mL^−1^, 100 µg·mL^−1^, 10 µg·mL^−1^ (black dots) were considered. WT cells are marked with a red dot. Results represent the mean from three independent experiments. The 95% confidence interval is shown. Correlation analysis of the data was performed using a Linear Correlation (Pearson r) test.

The analysis of the correlation rates between the observed antibacterial activity of the tested aqueous and ethanol extracts and the fatty acid (FA) content indicates that the SAFA fraction in the tested biomasses had an inhibitory effect on the growth of *Streptococcus pyogenes* PCM 2318 after the application of the water (r = −0.7135, *p* < 0.01) and ethanolic extracts (r = −0.5858, *p* < 0.05) at a concentration of 100 μg·mL^−1^ (Figure 3A,B).

**Figure 4:**
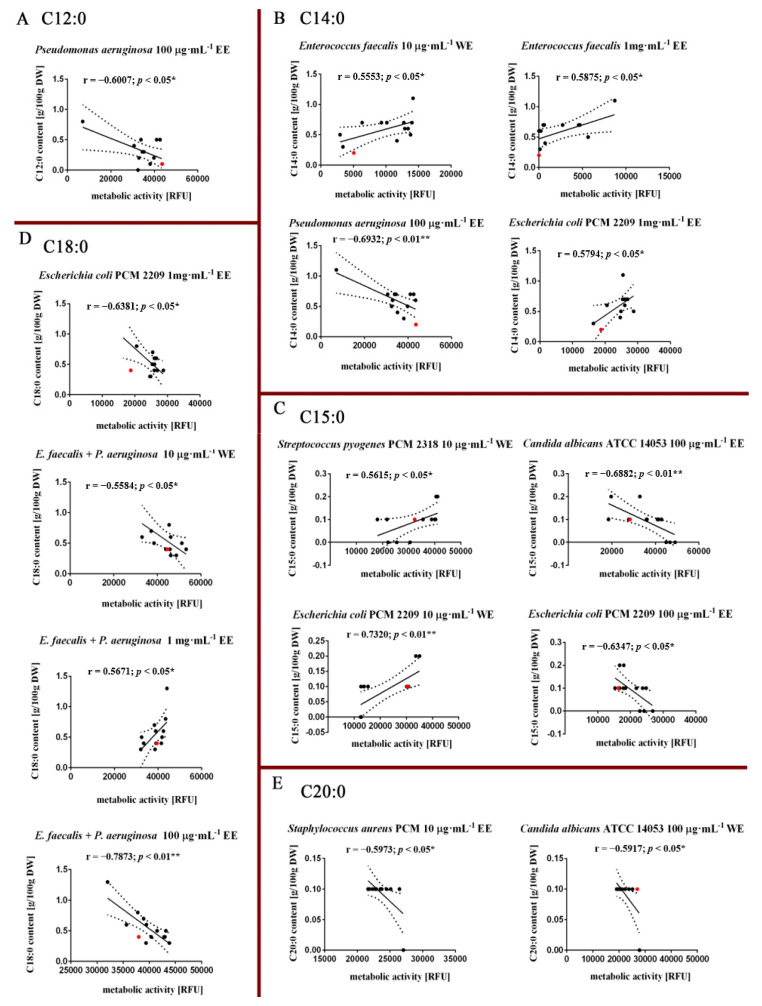
Fatty acid-mediated effect on the metabolic activity of selected bacterial species: *Streptococcus pyogenes* PCM 2318, *Staphylococcus aureus* PCM 458, *Pseudomonas aeruginosa*, *Escherichia coli* PCM 2209 and *Candida albicans* ATCC 14053. Correlation analysis between (**A**) Lauric acid (C12:0), (**B**) Myristic acid (C14:0), (**C**) Pentadecylic acid (C15:0), (**D**) Stearic acid (C18:0) and (**E**) Arachidic acid (C20:0) ratio and metabolic activity (RFU). Twelve CC-treated clones were considered (black dots). WT cells are indicated with a red dot. Results represent the mean from three independent experiments. The levels of fatty acids were calculated per g of dry weight. The 95% confidence interval is shown. Correlation analysis of the data was performed using a Linear Correlation (Pearson r) test. DW, dry weight.

However, the analysis of individual fatty acids in the profiles of the obtained biomasses of *P. nurekis* indicates that an increased proportion of lauric acid (C12:0), myristic acid (C14:0), pentadecanoic acid (C15:0) and stearic acid (C18:0) decreased statistically significantly the metabolic activity of the Gram-negative tested bacterial species, i.e., *Pseudomonas aeruginosa* and *Escherichia coli* PCM 2209, when ethanolic extracts were used at concentrations of 1 mg·mL^−1^ and 100 μg·mL^−1^ (Figure 4A–D). Interestingly, correlation analysis also revealed antifungal activity in the biomasses fraction characterized by an increased proportion of pentadecanoic acid (C15:0) as well as arachidic acid (C:20) in the FAs profile of *P. nurekis* strains when both water and ethanolic extracts were used at a concentration of 100 μg·mL^−1^ (Figure 4C,E). It was also noted that the increased content of myristic acid (C14:0), pentadecanoic acid (C15:0) and stearic acid (C18:0) in the FAs profile of the tested biomasses also stimulated the metabolic activity of *Enterococcus faecalis*, *Streptococcus pyogenes* PCM 2318, *Escherichia coli* PCM 2209 and in dual-species cultures—*E. faecalis* + *P. aeruginosa*—using ethanolic extracts at a concentration of 1 mg·mL^−1^ and water extracts at a concentration of 10 μg·mL^−1^ (Figure 4B–D).

**Figure 5:**
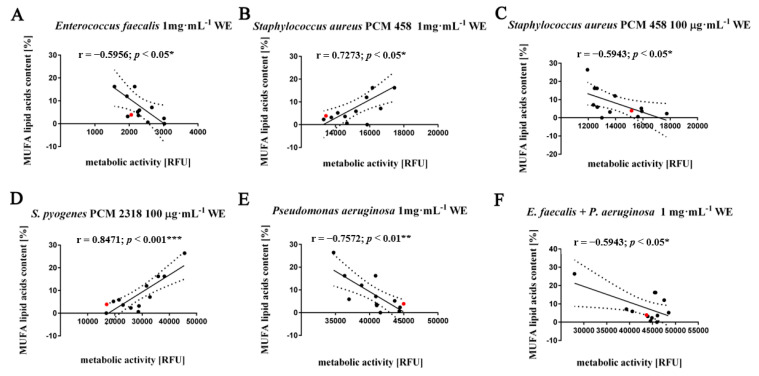
Effect of monounsaturated fatty acid (MUFA) (**A**–**F**) on the metabolic activity of selected bacterial species: *Enterococcus faecalis*, *Staphylococcus aureus* PCM 458, *Streptococcus pyogenes* PCM 2318, *Pseudomonas aeruginosa*, mixed two species culture—*Enterococcus faecalis* and *Pseudomonas aeruginosa*. Twelve water or ethanol extracts with defined concentrations of 1 mg·mL^−1^, 100 µg·mL^−1^ and 10 µg·mL^−1^ (black dots) were considered. WT cells are marked with a red dot. Results represent the mean from three independent experiments. The 95% confidence interval is shown. Correlation analysis of the data was performed using a Linear Correlation (Pearson r) test.

For total MUFA content, correlation rates analysis showed differential effects (stimulatory as well as inhibitory) of the water and ethanolic (WE, EE) extracts used. There was a statistically significant negative correlation between MUFA content and metabolic activity of *Staphylococcus aureus* PCM 458 after treatment with 100 μg·mL^−1^ water extracts (r = −0.5943, *p* < 0.05); *Enterococcus faecalis* (r = −0.5956, *p* < 0.05) and *Pseudomonas aeruginosa* after treatment with 1 mg·mL^−1^ water extracts (r = −0.7572, *p* < 0.01), and in a two-species culture: *Enterococcus faecalis* with *Pseudomonas aeruginosa* with the final water extract concentration 1 mg·mL^−1^ (r = −0.5943, *p* < 0.05) (Figure 5A,C,E,F). Interestingly, a stimulatory effect of MUFAs on cellular metabolic activity was observed in Gram-positive bacterial species, i.e., *Staphylococcus aureus* PCM 418, after treatment with water extracts at concentrations of 1 mg·mL^−1^ (r = 0.7273, *p* < 0.05), and *Streptococcus pyogenes* PCM 2318 after treatment with water extracts at concentrations of 100 μg·mL^−1^ (r = 0.8471, *p* < 0.001) (Figure 5B,D).

**Figure 6:**
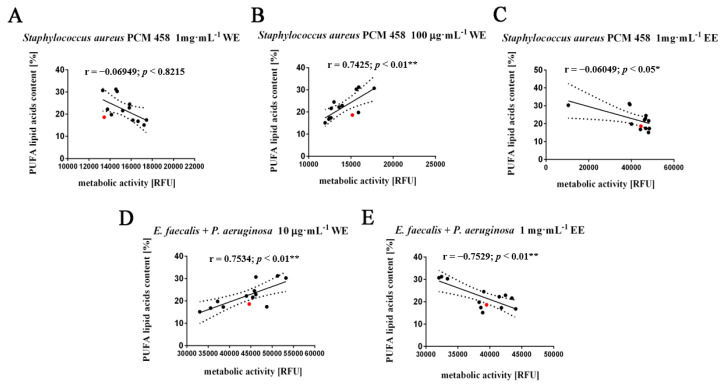
Effect of polyunsaturated fatty acid (PUFA) (**A**–**E**) on the metabolic activity of selected bacterial species: *Staphylococcus aureus* PCM 458, mixed two species cultures—*Enterococcus faecalis* and *Pseudomonas aeruginosa*. Twelve water or ethanol extracts with defined concentrations of 1 mg·mL^−1^, 100 µg·mL^−1^, 10 µg·mL^−1^ (black dots) were considered. WT cells are marked with a red dot. Results represent the mean from three independent experiments. The 95% confidence interval is shown. Correlation analysis of the data was performed using a Linear Correlation (Pearson r) test.

Among the considered fatty acids, the PUFA fraction showed an inhibitory effect on the metabolic activity of cells, mainly of the Gram-positive *Staphylococcus aureus* PCM 458, and a similar correlation was observed in the mixed culture of two species: *E. faecalis* with *P. aeruginosa* (Figure 6A,C,E). In addition, we noted a statistically significant positive correlation between the content of PUFAs and the increase in metabolic activity Gram-positive *Staphylococcus aureus* PCM 458 after treatment with 100 μg·mL^−1^ water extracts (r = 0.7425, *p* < 0.05), as well as in a mixed culture of two species: *E. faecalis* with *P. aeruginosa* with the lowest concentration of water extracts (r = 0.7525, *p* < 0.05) (Figure 6B,D).

The analysis of polyunsaturated fatty acids (PUFA) (sum of C18:2 linoleic acid, LA, omega 6) additionally showed increased metabolic activity of Gram-negative bacterium *Escherichia coli* PCM 2209 after treatment with ethanolic extracts at concentrations of 1 mg·mL^−1^ (r = 0.6757, *p* < 0.05) (not shown graphically).

The analysis confirmed the significant role of particular classes of fatty acids in inactivating the growth of selected species of Gram-positive bacteria (*Enterococcus faecalis*, *Streptococcus pyogenes* PCM 2318, *Staphylococcus aureus* PCM 458 and Gram-negative (*Pseudomonas aeruginosa* and *E. coli* PCM 2209) (Figure 4). Similarly, results obtained by Khoramnia, et al. (2013) indicated that the high fatty acid content plays an important role in broadening the antimicrobial spectrum of coconut oils rich in medium chain fatty acids (MCFA) obtained after fermentation using the Malaysian lipolytic yeast *Geotrichum candidum* [38]. The fatty acid concentrations obtained in the study by Cermak L. et al. (2015) on *P. nurekis* biomass are significantly lower than in the case of biomass obtained from polyploid clones *P. nurekis* subject of our analyses. These differences concern the content of myristic acid whose content in *P. nurekis* biomass is estimated at 1.74%, palmitic acid at 26.91% and linoleic acid (omega 6) at 4.13% [4]. In the case of biomasses obtained from polyploid clones, the content of these acids increases significantly to 5.1%, 61.1% and 31.2%, respectively (characteristics of the FA profile of biomasses are described in an earlier paper [15]). Additionally, the inhibitory activity of biomass on the growth of microorganisms in the work of Cermak L. et al. (2015) [4] was demonstrated at higher concentrations, i.e., 6 mg·mL^−1^ observed against *Escherichia coli*, *Salmonella enterica var. infantis K2*. In the case of our work, the antimicrobial concentrations of the applied extracts obtained from *P. nurekis* clone biomasses were relatively low (10 μg·mL^−1^ < 100 μg·mL^−1^ < 1 mg·mL^−1^). This clear difference could be explained by a difference in the composition or the amounts of the active FAs. The activity can also be variable with the target bacterium. Moreover, in his study, El Shoubaky et al. (2014) indicates that the antimicrobial potential of algae is closely correlated with the profile of saturated and unsaturated fatty acids and with a predominant fraction of myristic, palmitic, oleic and eicosapentaenoic acids (EPA) [39]. In other work, lauric acid, palmitoleic acid and long-chain polyunsaturated fatty acids (PUFAs) were found to be the most active, which confirms the effectiveness of *P. nurekis* biomass with increased lauric acid content in eliminating *Pseudomonas aeruginosa* cells [34,40]. In contrast, according to Al-Saif et al. (2014), the desired antimicrobial effect was characterized by algal extracts richest in palmitic acid > oleic acid > linoleic acid > myristic acid in concentration ranges of [43.7–75.5]% > [3.53–17.24]% > [0.6–16.56]% > [2.13–11.2]%, respectively [41]. However, based on the analysis of the correlation of the FA composition with the metabolic activity of microorganisms, the fatty acid profile of the studied *P. nurekis* biomass is as follows: linoleic acid (C18:2) [15.1–31.2]% > sums of oleic and elaidic acids (C18:1) [0–26.4]% > stearic acid (C18:0) [2.3–11.7]% > myristic acid (C14:0) [2.6–7.6]% > lauric acid (C12:00) [0.4–3.7]% > pentadecanoic acid (C15:0) [0.4–1.5]% > arachidic acid (C20:0) [0.4–1.5]%. Furthermore, palmitic acid has been assumed to be primarily responsible for the antibacterial activity of alga [31,41]. For the analyzed *P. nurekis* biomasses, the strongest negative correlation between palmitic acid level (C16:0) and metabolic activity was observed for *Streptococcus pyogenes* PCM 2318 (r = −0.329) and also for *Candida albicans* ATCC 14053 (r = −0.5161), but these correlations were of no statistical significance. The study of correlation coefficients between the observed antimicrobial activity and FA composition enabled the selection of lauric acid (C12:0), myristic acid (C14:0), and stearic acid (C18:0) as modulators of growth of mainly Gram-negative bacteria, i.e., *Pseudomonas aeruginosa* and *Escherichia coli* PCM 2209. On the other hand, pentadecanoic acid (C15:0), as well as arachidic acid (C20:0), are the main modulators of growth of *Candida albicans* ATCC 14053. Monounsaturated (MUFA), as well as polyunsaturated fatty acids (PUFA), are in most cases modulators of Gram-positive bacteria, i.e., *Enterococcus faecalis*, *Staphylococcus aureus* PCM 458. In the literature, reports that the antimicrobial efficacy of saturated FAs tends to decrease with the lengthening or shortening of the length of the carbon chain, with 10 or 12 carbon chains being the most active. In other studies, they report that FAs with 14, 16 or 18 carbon atoms may be stronger than FAs with 10 or 12 carbon atoms against certain species of bacteria, which also confirms our analysis [34,42]. Our results also confirm the analyses carried out by Zheng et al. (2005), who in their work indicate that long-chain PUFAs, such as oleic, linoleic and linolenic acids, are responsible for the antibacterial action of FAs, and that their mechanism of action is to inhibit the synthesis of bacterial FAs [43]. It has also been shown that long-chain unsaturated fatty acids, including linoleic acid, do not inhibit Gram-negative bacteria such as *E. coli*, which also confirmed our observations [43,44,45,46]. This demonstrated difference in sensitivity to fatty acids between Gram-positive and Gram-negative bacteria may be due to the impermeability of the outer membrane of Gram negative bacteria, which is an effective barrier to hydrophobic substances [43,47]. It is reported that the mechanism underlying the bactericidal action of the fatty acids of the extracts is not yet fully understood, but it seems that they promote destabilization of cell membranes, which eventually lead to leakage from the cell, besides limiting nutrient uptake and inhibiting cellular respiration [48].

In the case of anti-fungal activity, some FAs have been described as a potential factor against *Candida* sp. The most active ones include: short-chain saturated fatty acids (capric acid; mainly lauric acid), monounsaturated fatty acids (MUFA) (myristoleic and palmitoleic acid) and PUFAs (linoleic acid) [49]. The antifungal activity shown in our case, related to the presence of pentadecanoic acid (C15:0), as well as arachaidic acid (C20:0), may be due to their synergistic action with the others available in biomass FAs, as well as the other available bio-components; however, this would require further analysis. Interestingly, the synergistic actions of saturated fatty acids (mainly palmitic, myristic and stearic) monounsaturated ω-9 oleic, and polyunsaturated fatty acids (particularly arachidonic, dihomo-γ-linolenic and *cis*-11,14-eicosadienoic), as well as the presence in the biomass of some essential oils of brown seaweed *H. cuneiformis*, stimulated antifungal activity [50]. There are few literature reports on the composition of aqueous algae extracts, including the presence of fatty acids in them. Therefore, extraction at 100 °C for 30 min was used to increase the possibility of fatty acid penetration from the investigated biomasses of *Planktochlorella nurekis* strains. Khuwijitjaru et al. (2002) in their work indicate that the solubility of fatty acids with an even number of carbon atoms from 8 to 18 measured in the temperature range from 60 to 230 °C increased with temperature. The researchers also indicated that at temperatures higher than 160 °C, the logarithm of the solubility in mole fraction was linearly related to the reciprocal of the absolute temperature for each fatty acid, indicating that the water containing solubilized fatty acid molecules formed a regular solution at the higher temperatures [51]. Furthermore, in the PubChem Substance and Compound Databases, solubility values of the fatty acids most predominant in the *Planktochlorella nurekis* studied biomasses are provided, i.e., palmitic acid (C16:0), which shows a water solubility of 0.04 mg·mL^−1^ at 25 °C, and 1 g in 20 mL in ethanol; stearic acid (C18:0)—0.597 mg·mL^−1^ at 25 °C, 1 g in 20 mL in ethanol; linoleic acid LA, omega 6, (C18:2) showing a solubility in water of 1.59 mg·mL^−1^ at 25 °C; very soluble in ethanol; oleic acid (C18:1), omega 9—soluble in water of—1.15 × 10^−2^ mg·mL^−1^ at 25 °C, miscible with ethanol. It has also been pointed out in papers that obtaining other high value compounds (proteins and carbohydrates) in algae extracts is not always possible due to the incompatibility of their chemical structure and properties with the conditions related to the use of organic solvents, as well as other physical factors e.g., high extraction temperature. In contrast to hydrophobic compounds (fatty acids, hydrophobic pigments), hydrophilic compounds may undergo denaturation, precipitation or degradation during extraction in the most common organic solvents and also at higher temperatures, e.g., >40 °C [52,53]. Thus, it seems reasonable to conclude that in the extracts obtained in our study proteins/peptides cannot be treated as proper active ingredients/antimicrobial agents. 

It is also important to remember that algae extracts are mixtures not only of fatty acids, but also of other natural compounds (polysaccharides, polyphenolic compounds, pigments (including carotenoids, phycobiliproteins, chlorophylls); terpenoids, acrylic acid, steroids, halogenated ketones and alkanes, cyclic polysulphides, algae toxins, algaecides, plant-growth regulators) [5,54,55], and their antibacterial/antifungal activity is not only the result of the different actions of the individual components, but may be the result of their interaction, which may have a different effect on their overall effect.

### 2.3. Probiotic Efficiency of Planktochlorella Nurekis Extracts—Stimulating Growth of Lactic Acid Bacteria Lactobacillus rhamnosus ATCC 53103

Bioactive compounds of microalgae origin are not only a potential source of antibacterial, antifungal or antiviral activity. It turns out that the high structural diversity of polysaccharides in microalgae cells may be a valuable source of prebiotics stimulating the growth of specific strains of probiotic bacteria, including the *Lactobacillus* genus [56]. Therefore, in the next stage of the study, it was decided to check whether water and ethanolic extracts of *P. nurekis* can exert prebiotic effects on the growth of the probiotic species *Lactobacillus rhamnosus* ATCC 53103.

**Figure 7:**
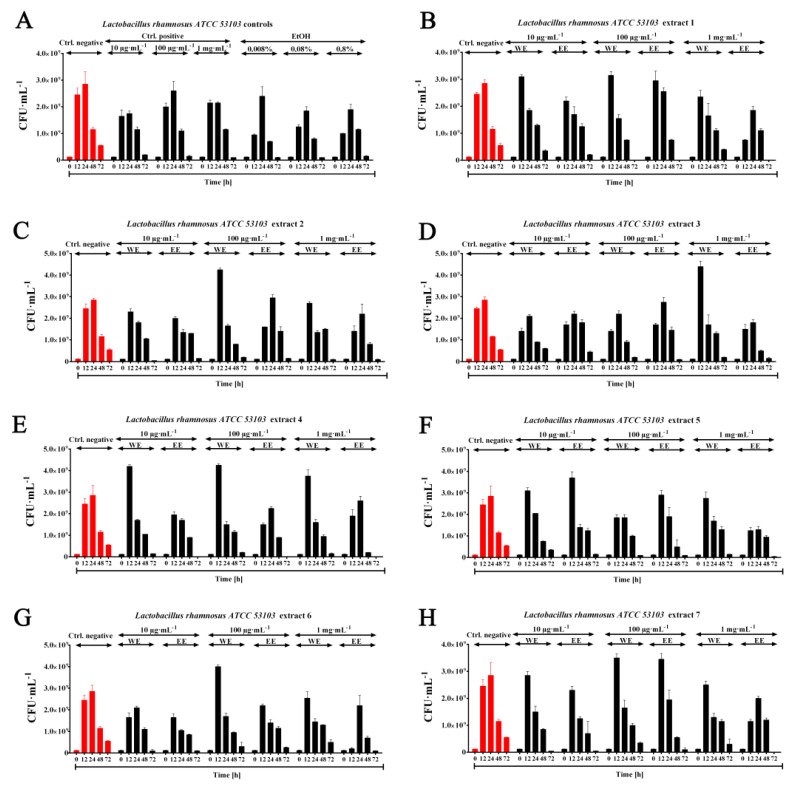

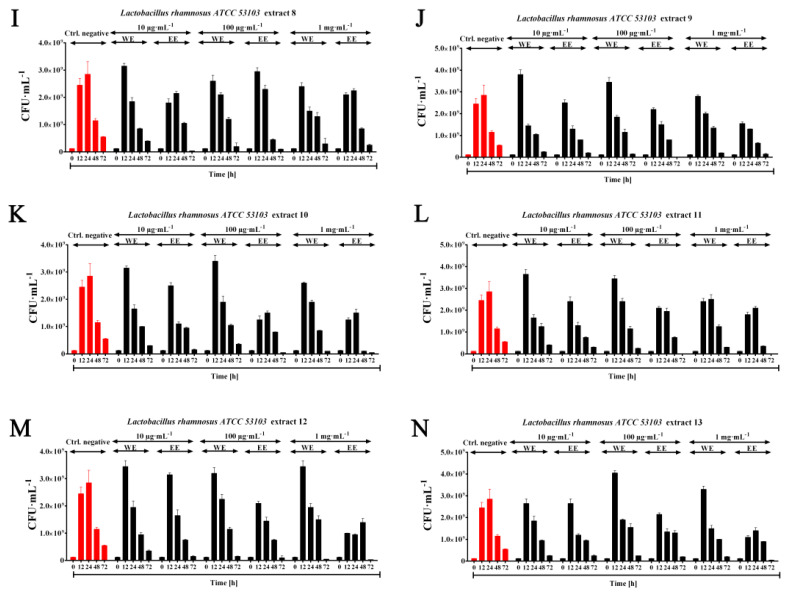
Stimulating effect of water and ethanolic extracts of algae (1–13, **A**–**N**) on the growth of probiotic species *Lactobacillus rhamnosus* ATCC 53103 (CFU·mL^−1^) after 12, 24, 48 and 72 h. MRS medium (without inulin addition) as a negative control and MRS medium with inulin addition with final concentrations—1 mg·mL^−1^, 100 μg·mL^−1^, 10 μg·mL^−1^—were used as positive control. For ethanol extracts, positive controls of appropriately diluted ethanol with final concentrations of—0.8%, 0.08%, 0.008% were also used.

The analysis showed a stimulating effect of water extracts 3 > 4 > 12 > 9 > 13 > 5 > 2 > 10 > 6 with a concentration of 1 mg·mL^−1^, 2 > 4 > 13 > 6 > 7 > 9 > 11 > 10 > 1 > 8 with a concentration of 100 μg·mL^−1^, 4 > 9 > 11 > 12 > 8 > 10 > 5 > 1 > 13 with a concentration of 10 μg·mL^−1^ on the growth of the probiotic species *Lactobacillus rhamnosus* ATCC 53103 in relation to the negative control, especially visible after 12 h of the conducted culture. In the case of ethanolic extracts, a stimulating effect was observed at a concentration of 100 μg·mL^−1^—extract 7 > 8 > 5, as well as at a concentration of 10 μg·mL^−1^—extract 5 > 12 > 13 (Figure 7). The decrease in CFU·mL^−1^ at the other measurement points (starting from 24 h) is due to the interaction on the cells of lactic acid accumulated in the medium and other metabolites limiting bacterial growth. However, the discrepancies in the results when assessing the effect of algal extracts on the growth of *Lactobacillus rhamnosus* using the OD optical density measurement method may be due to the fact that this technique also takes into account the presence of dead cells with a preserved integrity of cytoplasmic membranes, which does not fully reflect the physiological state of the culture (especially after the use of ethanolic extracts). Additionally, these discrepancies may be a consequence of cells settling to the bottom of the culture plate, despite shaking the culture during incubation (results not shown). In the work of Bhowmik et al. (2009), the stimulatory effect of *Spirulina platensis* dry mass was studied on three lactic acid bacteria *Lactobacillus casei* MTCC 1423, *Lactobacillus acidophilus* MTCC 447, *Streptococcus thermophilus* MTCC 1938. The addition of dry biomass of *S. platensis* at concentrations of 10 mg·mL^−1^ promoted the growth of *Lactobacillus acidophilus* to 171.67% and *S. thermophilus* 185.84%, respectively. At a concentration of 1 mg·mL^−1^, growth stimulation of 63.93% was observed for *Lactobacillus casei* MTCC 1423 after 10 h of incubation [57]. Prebiotic effects of tested extracts of biochemically different biomasses of *P. nurekis* on growth *Lactobacillus rhamnosus* ATCC 53103 could be observed at lower concentrations of water extracts—100 µg·mL^−1^ by 73% and 10 µg·mL^−1^ by 51% respectively after 12 h of incubation. At the concentration used by the author of the publication, i.e., 1 mg·mL^−1^, an increase of 80% was observed compared to the control not treated with water extract.

Thus, due to the high cost of producing prebiotics, oligo- and polysaccharides extracted from marine and freshwater algae may provide an alternative source of prebiotics to stimulate the growth of strains included in the intestinal microbiota (*Lactobacillus* and *Bifidobacterium* spp.)

### 2.4. Absence of Synergism and Antagonism between Selected Groups of Bacteria in the Presence of Algal Extracts

During the study it was assumed that the mixed bacterial culture shows synergism when it achieves a significantly higher metabolic activity measured by the resazurin test compared to the mixed negative control culture (without the addition of extracts). The analyses showed no differences in the effect of extracts (both water and ethanolic 1–13) on the increase in metabolic activity of the mixed bacterial culture compared to the negative control. In addition, no zones of inhibition were observed at the site of culture crossings between the different bacterial species, indicating the antagonistic properties of the tested bacterial species (results not shown).

## 3. Materials and Methods

### 3.1. Microalgal Extracts Preparation

Twelve clones of the microalga *Planktochlorella nurekis* with improved biochemical features were used. A detailed description of biochemical profiles of modified microalgal clones can be found elsewhere [15]. Briefly, a wet biomass of *Planktochlorella nurekis* strains (1–13) was obtained after cell culture centrifugation (3000× *g*, 5 min), which was then subjected to thin layer drying at 42 °C for 24 h using a moisture analyzer. To prepare the microalgal extracts of twelve clones of the microalga *Planktochlorella nurekis* and one control clone, two solvents were considered, namely water and 80% ethanol. To obtain water extracts (WE), 100 mg of microalgal dry weight were added to sterile ultra pure water to give the stock concentration of 100 mg·mL^−1^. The resulting suspensions were boiled at 100 °C for 30 min. In the next step, the resulting suspension was centrifuged at 12,000 RPM for 10 min and the supernatant was transferred to new eppendorf tubes. Supernatants were collected and stored until use at −20 °C. To obtain ethanolic extracts (EE), 100 mg of microalgal dry weight were added to 80% ethanol to give the stock concentration of 100 mg·mL^−1^. The samples were then incubated at 37 °C for 24 h with shaking (1000 RPM) and centrifuged (12,000 RPM, RT, 10 min). Supernatants were collected and stored until use at −20 °C [58].

### 3.2. Bacterial Strains and Antimicrobial Potential of Algal Extracts against Selected Microorganisms

The antimicrobial potency of each microalgal extract was evaluated using the following microorganisms: three strains of Gram-positive (*Enterococcus faecalis*, *Staphylococcus aureus* PCM 458, *Streptococcus pyogenes* PCM 2318), two strains of Gram-negative (*Pseudomonas aeruginosa*, *Escherichia coli* PCM 2209) and mixed cultures from 2 species: *Enterococcus faecalis* and *Pseudomonas aeruginosa*. Additionally, the antifungal activity of the tested extracts was checked using *Candida albicans* ATCC 14053 strain. Clinical strains of *Pseudomonas aeruginosa*, *Enterococcus faecalis* were received from the collections of microorganisms of the Frederic Chopin Provincial Specialist Hospital in Rzeszów, Poland which were obtained during routine diagnostic cultures. 

The microorganisms were cultured on Nutrient Agar NA (meat extract—10 g·L^−1^, peptone—10 g·L^−1^, sodium chloride—5 g·L^−1^, agar—20 g·L^−1^, pH—7.0) and on YPD medium (yeast extract—10 g·L^−1^, peptone—20 g·L^−1^, glucose—20 g·L^−1^, pH—7.2). The water and ethanolic extracts (WE, EE) were used to prepare dilutions, i.e., 1:10, 1:100, with which the tested microorganism species were treated. Antibacterial and growth inhibitory properties of algal extracts against selected microorganisms were tested using a spot-on-lawn method. Briefly, microorganism assay plates were prepared whereby the tested bacteria and yeast (8-log CFU·mL^−1^) were seeded. The microorganism-seeded assay plates were marked in thirteen pie-section quadrants and 5 μL of each algal extract dilution was surface-spotted onto the indicator lawn. In the case of ethanol extracts, positive controls of appropriately diluted ethanol, i.e., 80%, 8%, 0.8%, also surfaced spotted onto the indicator lawn. Plates were allowed to incubate overnight. Growth inhibition was evaluated after incubation (24 h at 37 °C—bacteria and 29 °C—yeast) by observations of the zone of inhibition around the spots with the test organism and were recorded using a G:BOX SYNGENE gel doc system with high resolution camera.

### 3.3. Fatty Acids Composition Analysis 

The fatty acids in the biomass of *Planktochlorella nurekis* strains were determined as described elsewhere [15].

### 3.4. Growth Inhibition Assay—Effect of Water and Ethanolic Extracts of Microalgae (WE, EE) on Metabolic Activity of Tested Microorganisms in Liquid Cultures 

The antimicrobial properties of water and ethanol extracts of microalgae (WE, EE) were determined using the Alamar Blue (resazurin) cell viability assay according to Schneemann et al. [59] The principle of this method is based on the irreversible reduction of blue dye resazurin to red-fluorescent resorufin only by metabolically active cells. Briefly, overnight cultures of tested microorganisms in LB medium (tryptone—10 g·L^−1^, yeast extract—5 g·L^−1^, sodium chloride—5 g·L^−1^, pH—7.0) (bacteria), and YPD medium (yeast extract—10 g·L^−1^, peptone—20 g·L^−1^, glucose—20 g·L^−1^, pH—7.2) (yeasts) were diluted to an optical density of 0.02 to 0.05 at 600 nm. Water and ethanolic extracts of algae were added to the prepared cultures to obtain final concentrations of—1 mg·mL^−1^, 100 µg·mL^−1^ and 10 µg·mL^−1^ (starting extracts concentration solutions for analysis were 100 mg·mL^−1^). The cultures were then dispensed at 150 µl per 96-well microplate in triplicate and incubated for 24 h at 37 °C (bacteria) and 29 °C (yeasts) with 600 RPM shaking. In addition, a negative control (no compound) and a positive control were prepared for each culture with the addition of chloramphenicol 100 μg·mL^−1^ culture for bacteria and 1 mg·mL^−1^ cycloheximide culture for *Candida albicans* strain. For ethanolic extracts, positive controls of appropriately diluted ethanol reaching final concentrations in samples of—0.8%, 0.08% and 0.008% were also used. After 24 h of incubation, 10 µL of resazurin solution (0.2 mg·mL^−1^ in PBS) was added to each well of the 96-well plate and incubated for 2 h (until the negative control changed color from blue to red/pink). To assess the impact of the analyzed algae extracts on the metabolic activity of tested microorganisms, a fluorescence measurement at 560 nm after excitation at 590 nm was taken.

### 3.5. Prebiotic Effect of Water and Ethanolic Algal Extracts (WE, EE) on the Growth of the Probiotic Species Lactobacillus rhamnosus ATCC 53103 

Overnight culture of *Lactobacillus rhamnosus* ATCC 53103 in Man, Rogosa and Sharpe (MRS) medium (BTL, Łódź, Poland) was standardized to an optical density of 0.02 at 600 nm. Water and ethanolic algal extracts were added to the prepared cultures to obtain final concentrations of 1 mg·mL^−1^, 100 µg·mL^−1^ and 10 µg·mL^−1^ (starting extract concentration solutions for analysis were 100 mg·mL^−1^). The cultures were then dispensed at 300 µL per 96-well microplate in triplicate and incubated at 37 °C. Optical density measurements were taken every 2 h during 12 h incubation, and then after 24 h, 48 h and 72 h of culture to determine the time at which bacterial growth of the probiotic species would be significantly increased. A negative control without inulin and a positive control with inulin were prepared for each culture with final concentrations of 1 mg·mL^−1^, 100 μg·mL^−1^, 10 μg·mL^−1^. In the case of ethanolic extracts, positive controls of appropriately diluted ethanol reaching final concentrations in samples (0.8%, 0.08%, 0.008%) were also used. The effect of algal extracts on the *Lactobacillus rhamnosus* ATCC 53103 kinetics growth was determined by measuring the optical density of the culture at OD 600 nm using an Infinite M200 analyzer (Tecan, Switzerland) and by counting colonies from appropriate dilutions of the culture on MRS agar. The results were expressed in CFU·mL^−1^.

### 3.6. Determination of the Effect of Algal Extracts on the Synergistic Growth of a 2 Species Culture of Gram (+/+), Gram (−/−), Gram (+/−) Bacteria Using a Resazurin Assay

The following combinations of bacterial species were used to analyze the effect of the obtained algal extracts (WE, EE) on bacterial interactions in mixed cultures:

- Gram-negative (−) with Gram-positive (+) (*Escherichia coli* PCM 2209 + *Enterococcus faecalis*)
- Gram-positive (+) with Gram-positive (+) (*Staphylococcus aureus* PCM 458 + *Streptococcus pyogenes* PCM 2318)
- Gram-negative (−) with Gram-negative (−) (*Escherichia coli* PCM 2209 + *Pseudomonas aeruginosa*)

Briefly, overnight cultures of bacteria incubated at 37 °C in LB medium were standardized to an optical density of 0.02 to 0.05 at 600 nm and then mixed in a ratio of 1:1. Water and ethanolic extracts of algae were added to the cultures thus prepared according to the methodology described in the section on the prebiotic effect of water and ethanolic algal extracts (WE, EE) on the growth of the probiotic species *Lactobacillus rhamnosus* ATCC 53103. 

### 3.7. Effect of Water (WE) and Ethanolic (EE) Algal Extracts P. nurekis on a Microbial Antagonism

The influence of the analyzed extracts on the antagonism between the tested bacterial species *Escherichia coli* PCM 2209, *Enterococcus faecalis*, *Staphylococcus aureus* PCM 458, *Streptococcus pyogenes* PCM 2318, *Pseudomonas aeruginosa* and mixed cultures from two species—*Enterococcus faecalis* and *Pseudomonas aeruginosa*—was carried out on the LB agar medium using the cross streak method. For this purpose, overnight cultures of all analyzed bacterial species were standardized to an optical density of 0.02 at 600 nm wavelengths. Thus, prepared cultures were seeded on LB agar plates (BTL, Łódź, Poland) with the addition of algae water and ethanolic extracts with final concentrations of 1 mg·mL^−1^, 100 µg·mL^−1^ and 10 µg·mL^−1^. The antimicrobial activity of the tested microorganisms was checked by seeding cultures of one of the analyzed species by a single streak in the center of the agar plate and then, after their absorption into the medium, the plate is seeded with the microorganisms tested by a single streak perpendicular to the central streak. Plates with bacterial cultures were incubated at 37 °C for 24 h and the antimicrobial interactions are analyzed by observing the inhibition zone size in comparison with the control plate (LB agar without algal extracts).

### 3.8. Statistical Analysis

The mean values ± SD were calculated on the basis of at least three independent experiments. Correlation analysis of the data was performed using a Linear Correlation (Pearson r) test. A *p*-value < 0.05 was considered statistically significant.

## 4. Conclusions

We analyzed for the first time the antimicrobial activity of extracts obtained from the biomass of twelve clones of the *Planktochlorella nurekis* microalgae with improved biochemical characteristics compared to their unmodified counterpart. The obtained extracts with a specific composition of biocompounds modulate the growth of bacteria of the species *Lactobacillus rhamnosus*, *Enterococcus faecalis*, *Staphylocoocus aureus*, *Pseudomonas aeruginosa*, *Escherichia coli* and *Candida albicans*. The study of correlation coefficients between the observed antimicrobial activity and FA composition enabled the selection of lauric acid (C12:0), myristic acid (C14:0), and stearic acid (C18:0) as modulators of growth of mainly Gram-negative bacteria, i.e., *Pseudomonas aeruginosa* and *Escherichia coli* PCM 2209. On the other hand, pentadecanoic acid (C15:0), as well as arachidic acid (C20:0), are the main modulators of growth of *Candida albicans* ATCC 14053. Monounsaturated (MUFA) as well as polyunsaturated fatty acids (PUFA), are in most cases modulators of Gram-positive bacteria, i.e., *Enterococcus faecalis* and *Staphylococcus aureus* PCM 458. Given the broad range of pathogens that are inhibited, these obtained biomasses may produce compounds that could prove promising candidates for potentially novel antibiotic and antifungal drugs.

## Data Availability

The data presented in this study are available on request from the corresponding author.

## Sample Availability

Samples of the of algal biomasses are not available from the authors.
